# Bending Properties of Standardized Photopolymer–Silicone Hybrid Structures Manufactured via PolyJet Matrix

**DOI:** 10.3390/ma18245612

**Published:** 2025-12-14

**Authors:** Mateusz Rudnik, Wiktor Szot, Natalia Kowalska, Paweł Szczygieł

**Affiliations:** Faculty of Mechatronics and Mechanical Engineering, Kielce University of Technology, Tysiąclecia Państwa Polskiego 7 Ave., 25-314 Kielce, Poland; wszot@tu.kielce.pl (W.S.); nkowalska@tu.kielce.pl (N.K.); pszczygiel@tu.kielce.pl (P.S.)

**Keywords:** PolyJet Matrix, photopolymer resin, silicone filling, cellular structures, hybrid specimens, three-point bending, static bending test, mechanical characterization, additive manufacturing, polymer composites

## Abstract

The study presented an analysis of the behaviour of cellular structures under bending, produced using the PolyJet Matrix (PJM) additive manufacturing method with photopolymer resin. Structures with regular cell geometry were designed to achieve a balance between stiffness, weight reduction, and energy absorption capacity. The aim of this study was to investigate the influence of unit-cell topology (quasi-similar, spiral, hexagonal honeycomb, and their core–skin hybrid combinations) on the flexural properties and deformation mechanisms of PolyJet-printed photopolymer beams under three-point bending. Additionally, all cellular configurations were fully infiltrated with a low-modulus platinum-cure silicone to evaluate the effect of complete polymer–elastomer interpenetration on load-bearing capacity, stiffness, ductility, and energy absorption. All tests were performed according to bending standard on specimens fabricated using a Stratasys Objet Connex350 printer with RGD720 photopolymer at 16 µm layer thickness. The results showed that the dominant failure mechanism was local buckling and gradual collapse of the cell walls. Among the silicone-filled cellular beams, the QS-Silicone configuration exhibited the best overall flexural performance, achieving a mean peak load of 37.7 ± 4.2 N, mid-span deflection at peak load of 11.4 ± 1.1 mm, and absorbed energy to peak load of 0.43 ± 0.06 J. This hybrid core–skin design (quasi-similar core + spiral skin) provided the optimum compromise between load-bearing capacity and deformation capacity within the infiltrated series. In contrast, the fully dense solid reference reached a significantly higher peak load of 136.6 ± 10.2 N, but failed in a brittle manner at only ~3 mm deflection, characteristic of UV-cured rigid photopolymers. All open-cell silicone-filled lattices displayed pseudo-ductile behaviour with extended post-peak softening, enabled by large-scale elastic buckling and silicone deformation and progressive buckling of the thin photopolymer struts. The results provided a foundation for optimising the geometry and material composition of photopolymer–silicone hybrid structures for lightweight applications with controlled stiffness-to-weight ratios.

## 1. Introduction

Cellular structures produced by additive manufacturing methods are the subject of systematic research in the field of materials mechanics and structural engineering [1,2]. Their properties result from the geometry of the cells and the way they are connected, which allows the deformation and energy characteristics to be shaped without changing the material composition [3,4,5,6,7]. Depending on their topology, these structures can exhibit conventional, auxetic or gradient behaviour, and their mechanics are based on the complex interaction between bending, compression and buckling of the cell elements [8,9,10].

Three-dimensional printing using photopolymer resins enables the production of structures with small cell sizes and high dimensional accuracy [11,12,13,14]. In comparison to extrusion methods, photopolymerisation ensures uniform cross-sections and a smooth surface, which reduces the impact of interlayer discontinuities on mechanical behaviour [15,16,17,18]. However, these types of materials are brittle and susceptible to defects, so their behaviour under bending loads requires separate analysis with regard to cell topology, print orientation and curing conditions [19,20,21].

Flexural deformations of cell ribs dominate in open structures, while in structures with lower porosity, interaction with local compression and buckling of walls occurs. Analysis of load-deflection curves allows the degree of anisotropy and the role of layer orientation in shaping the mechanical response to be determined [22,23,24,25].

The objective of this study is to evaluate the effect of cell geometry and printing orientation on the bending performance of cellular structures fabricated from photopolymer resin. This study systematically evaluates the flexural response of PolyJet-printed RGD720 photopolymer beams under three-point bending [26] focusing on the effect of unit-cell topology and complete infiltration with a soft platinum-cured silicone (Shore 30A). The key novelty lies in the fabrication and testing of fully interpenetrating photopolymer–silicone hybrid cellular structures—an approach that enables the coexistence of rigid load-bearing struts and a highly compliant continuous elastomeric phase, closely mimicking the mechanical behaviour of biological soft tissues such as cartilage, meniscus, or intervertebral discs [27]. The recorded load–deflection curves and derived parameters (peak load, deflection at peak, absorbed energy, and post-peak ductility) provide quantitative insight into how core–skin lattice architecture governs the transition from brittle photopolymer-dominated response to pseudo-ductile, tissue-like behaviour dominated by large-scale silicone deformation.

## 2. Materials and Methods

### 2.1. PolyJet Matrix Technology

The principle behind this technology is similar to that of 2D printers. As with inkjet printers, print heads spray successive layers of liquid photo-curable polymer resin onto the work platform, which is then cured by ultraviolet (UV) light. PolyJet technology was developed by Objet (now Stratasys). Currently, in its Triple-Jet version, it is considered one of the most advanced and multi-material technologies for processing photopolymers.

The entire printing process consists of four consecutive stages, where the resin material is heated to a temperature of 30–75 °C, which allows for favourable viscosity. Next, print heads moving directly above the working platform spray the heated resin, thus creating the first layer of material. In addition, UV lamps located on the print T-head cure the previously applied material, resulting in a finished fragment (layer) of the printed part. After completing the laying and construction of a given layer of the element, the working platform lowers by a value corresponding to its height, and the entire process is repeated until the finished model is obtained.

Components printed using PolyJet Matrix technology (Stratasys corp. Rehovot, Israel)are characterised by high accuracy and a very smooth surface finish. Another significant advantage is the ability to build models from a wide range of hard, flexible and transparent materials. A characteristic feature of PolyJet technology, unlike other rapid prototyping methods, is the ability to apply material continuously from different groups of print heads. This type of solution allows for control over the amount of material dispensed. In addition, each layer of material applied is fully cross-linked and exposed, which, unlike other methods using liquid photopolymers, eliminates the need for re-exposure of the part after printing.

**Figure 1:**
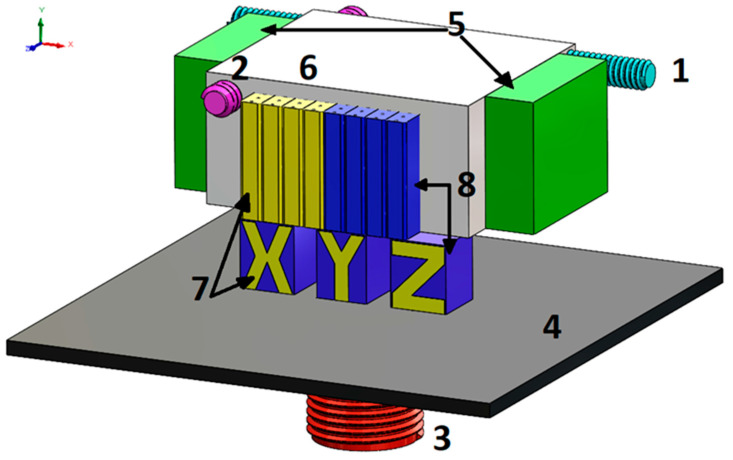
Components of a printer implementing PolyJet Matrix technology: 1—OX axis (movement of the head block); 2—OY axis (movement of the head block); 3—OZ axis (vertical movement of the working platform); 4—Build platform; 5—UV lamps; 6—Head block; 7—Support material; 8—Model material [28,29].

The Connex 350 printer with PJM technology uses a head block (6) that moves along guides along the OX (1) and OY (2) axes. The model material (8) and support material (7) are sprayed layer by layer with specified accuracy onto the working platform (4), which lowers with each layer applied along the guide on the OZ axis (3). Each layer of the model and support material is cured with light using UV lamps (5) located on both sides of the head block, as shown in the diagram below (Figure 1).

### 2.2. Materials

RGD720 is a rigid, translucent photopolymer used in PolyJet additive manufacturing. It provides dimensional stability and allows fabrication of thin-walled structures and complex geometries with a layer resolution of ~0.1 mm. The material exhibits a tensile strength of 50–60 MPa, elongation at break of 10–20%, flexural modulus of 2000–2500 MPa, Shore D hardness of 75–85, water absorption below 0.5% (24 h), and a heat deflection temperature of 45–50 °C at 0.45 MPa. RGD720 can be combined with other PolyJet resins to adjust mechanical properties and translucency [30].

Siliform 25 is a two-component RTV-2 silicone rubber (mixing ratio 100:3 by weight) with a pot life of 30–40 min and full cure after ~24 h at room temperature (20–23 °C). The cured material shows Shore A hardness of 25, elongation at break of ~600%, tear strength of 3.8 N/mm, viscosity of 19,000 mPa·s, and linear shrinkage below 0.3%. It is chemically and thermally stable and is commonly applied for elastic mould fabrication and casting of polymer systems such as epoxy or polyurethane resins [31].

### 2.3. Samples Preparation

Three types of cellular structures marked as H, S and Q were selected. These structures were incorporated into standardised mouldings to assess their impact on the mechanical properties of the samples. The samples were printed using PolyJet Matrix technology on a Connex 350 printer in High Quality (HQ) mode, which ensured high quality and precision. The layer height was 16 µm, which allowed for very accurate structural reproduction and minimisation of surface defects. The tests provide important data on the mechanical properties of selected cellular structures depending on the printing angle and the type of cellular structure used.

**Figure 2:**
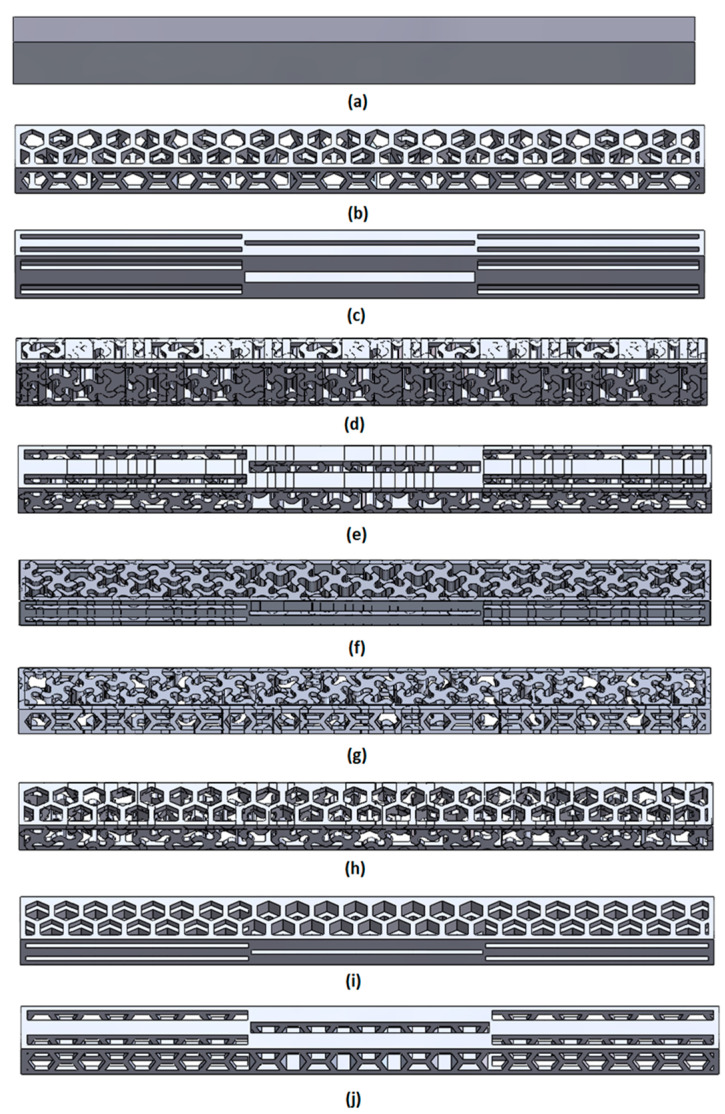
(**a**) Solid (fully dense reference); (**b**) HH—homogeneous hexagonal honeycomb; (**c**) QQ—homogeneous quasi-similar lattice; (**d**) SS—homogeneous spiral lattice; (**e**) QS—quasi-similar core with spiral skin layers; (**f**) SQ—spiral core with quasi-similar skin layers; (**g**) SH—spiral core with hexagonal skin layers; (**h**) HS—hexagonal core with spiral skin layers; (**i**) HQ—hexagonal core with quasi-similar skin layers; (**j**) QH—quasi-similar core with hexagonal skin layers.

These results can be used to optimise 3D printing processes and design materials with defined mechanical properties, which has wide applications in various industries. RGD720, a photopolymer resin, was used to make the samples. For three-point bending tests in accordance with bending standard, standard beams and beams with combinations of cellular structures were manufactured, designated as:

FF (full), SS (spiral), HH (hexagonal), QQ (quasi-self-similar), SH (spiral–hexagonal), SQ (spiral–quasi-self-similar), HS (hexagonal–spiral), HQ (hexagonal–quasi-self-similar), QS (quasi-self-similar–spiral) and QH (quasi-self-similar–hexagonal). In selected cases, the cellular structures in the beams were filled with moulding silicone to examine the effect of filling on mechanical properties. The samples had dimensions Lb = 120 mm, h = 10 mm, b = 6 mm and included 10 different structural configurations: FF, HH, QQ, SS, HS, HQ, QS, QH, SH, SQ. In this designation, the first letter refers to the structure inscribed in a wall with dimensions of 10 mm × 120 mm, and the second letter refers to the structure inscribed in a wall with dimensions of 6 mm × 120 mm. FF stands for a full sample, H stands for a hexagonal structure, S stands for a spiral structure, and Q stands for a quasi-self-similar structure (Figure 2).

Three-point bending tests were performed in accordance with bending standard using an Inspekt Mini 3 kN universal testing machine (Hegewald & Peschke, Nossen, Germany) equipped with a force sensor capable of precise load acquisition throughout the entire deformation range. The specimens (120 mm × 10 mm × 6 mm) were prepared following the geometry recommended for polymeric structural materials. To ensure repeatable support conditions, each sample was embedded in RTV silicone placed inside a MEX-fabricated form, which minimized local surface-contact irregularities during initial loading. The support span was set according to the standard requirement M = 16 h, resulting in a span of 96 mm for a specimen thickness of 6 mm. The bending fixture was driven at a crosshead speed of 2 mm/min, consistent with quasi-static loading conditions prescribed for polymer materials. Continuous force–deflection acquisition was performed over the entire loading range until the end of the experiment, which was terminated at a displacement of 25 mm, regardless of the moment of failure [27].

The samples were produced using PolyJet Matrix technology, which enables the precise formation of various cellular structures within the material. PJM technology allows for the creation of complex geometries that can influence the mechanical properties of the material, such as flexural strength, stiffness and fracture resistance. Each of the ten sample configurations (FF, HH, QQ, SS, HS, HQ, QS, QH, SH, SQ) was tested under conditions compliant with ISO 178. Specifically: FF samples were solid samples with no internal cellular structures; HH samples had a hexagonal structure inscribed in a 10 mm × 120 mm wall and a second hexagonal structure inscribed in a 6 mm × 120 mm wall; QQ samples had a Quasi-Self-Similar structure inscribed in both walls; SS samples had a spiral structure in both walls; HS samples had a hexagonal structure in a wall measuring 10 mm × 120 mm and a spiral structure in a wall measuring 6 mm × 120 mm; the HQ, QS, QH, SH, and SQ samples had combinations of hexagonal (H), spiral (S), and quasi-self-similar (Q) structures in both walls, respectively. The volume of the FF beam is 7.20 cm^3^, the HH beam—1.98 cm^3^, the HQ beam—2.57 cm^3^, the HS beam beams is 2.81 cm^3^, QH beams—2.55 cm^3^, QQ beams—3.37 cm^3^, QS beams—3.61 cm^3^, SH beams—2.74 cm^3^, SQ beams—3.55 cm^3^, while the largest volume was obtained for SS beams, amounting to 7.11 cm^3^. Each sample was subjected to three-point bending, and the results were recorded and analysed. These tests allow the assessment of the influence of different cell structures on the bending properties of the material, which is important in the design and optimisation of materials for various engineering applications.

All specimens had final dimensions of 120 mm × 10 mm × 6 mm (length × width × height) and were manufactured in accordance with ISO 178 (Plastics—Determination of flexural properties). The beams were printed on a Stratasys Objet Connex350 PolyJet printer using rigid transparent photopolymer RGD720 as the model material and SUP705 as soluble support. Printing was carried out in High Quality mode with a layer thickness of 16 µm, matte surface finish, and full UV post-curing after support removal. Ten identical replicates were produced for each configuration.

A straightforward designation system was used. The code consists of one or two capital letters followed, when applicable, by the suffix “-Silicone”:

- FF—fully dense reference beam (no lattice);
- QQ—homogeneous quasi-similar lattice throughout the entire 6 mm height;
- SS—homogeneous spiral lattice throughout the entire 6 mm height;
- HH—homogeneous hexagonal honeycomb throughout the entire 6 mm height;
- QS—quasi-similar lattice in the central zone + spiral lattice in the top and bottom skin layers;
- SQ—spiral lattice in the central zone + quasi-similar lattice in the top and bottom skin layers;
- QH—quasi-similar central zone + hexagonal skin layers;
- HQ—hexagonal central zone + quasi-similar skin layers;
- SH—spiral central zone + hexagonal skin layers;
- HS—hexagonal central zone + spiral skin layers.

## 3. Results

This section presents the mechanical response of the samples under three-point bending, including load–displacement behaviour, failure modes, and comparative analysis of structure types.

**Figure 3:**
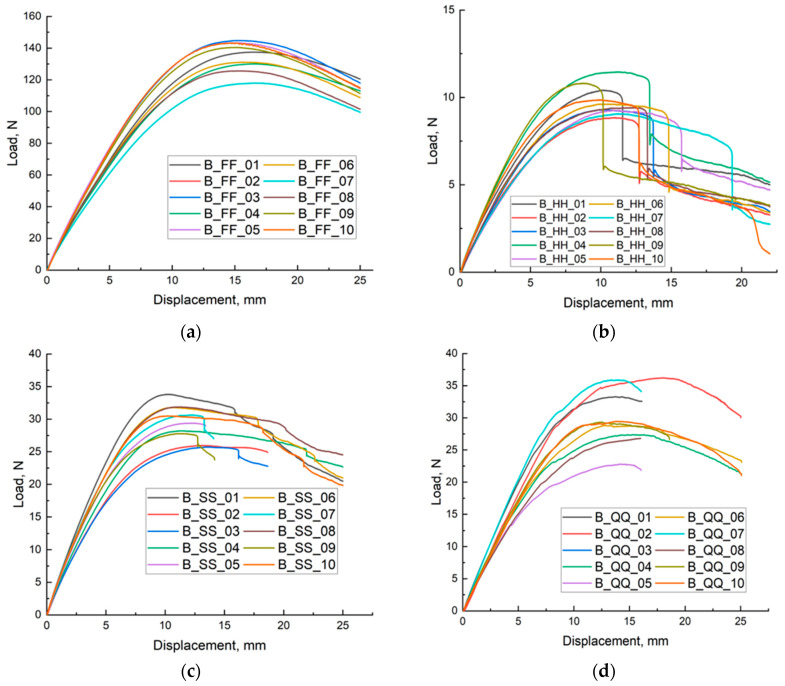
Load–Displacement plots for three-point bending for models manufactured from RGD 720 material with the following structures: (**a**) RGD 720; (**b**) FF; (**c**) SS; (**d**) QQ.

Figure 3a presents the load–displacement curves recorded during three-point bending tests of ten fully dense (FF) specimens fabricated from the reference material. The curves exhibit exceptional mutual congruence and reproducibility, as evidenced by their near-perfect superposition throughout the entire deformation history. Each curve displays the characteristic response of a homogeneous, dense solid: an extended linear-elastic regime with high apparent bending stiffness, a well-defined maximum load (F_max_) marking the onset of macroscopic damage, followed by pronounced post-peak softening with a prolonged, gradual load decay that signifies substantial fracture process zone development and considerable toughness.

Statistical analysis of the peak load values yields a mean F_max_ of 136.55 ± 10.16 N (mean ± SD), corresponding to a coefficient of variation (CV) of 7.4%. The maximum observed value was 151.32 N, while the minimum was 117. Concurrent displacement at peak load (L_max_) showed even greater consistency, with a mean of 15.78 ± 0.68 mm (CV ≈ 4.3%), and individual values confined to a narrow interval between 15.10 mm and 16.66 mm. The remarkably low scatter in both critical mechanical parameters—both in terms of absolute force and the associated mid-span deflection—confirms the microstructural uniformity and minimal batch-to-batch variability of the fully dense material. The extended post-peak plateau and controlled load reduction observed consistently across all specimens further indicate significant energy dissipation capacity through distributed microcracking and plastic deformation prior to catastrophic failure. Consequently, the dataset presented in Figure 3a, supported by the tight statistical distribution of F_max_ and L_max_, constitutes a highly reliable reference baseline for the material in its fully dense state under three-point bending. This benchmark enables rigorous, quantitative evaluation of the mechanical penalties—or potential benefits—introduced by deliberate porosity or cellular architectures manufactured from the same base material and tested under identical loading and boundary conditions. The fully solid configuration thus represents the upper performance envelope in terms of bending strength, stiffness, and load-bearing capacity against which all lightweight or architecture variants can be systematically compared.

In Figure 3b the force–deflection behaviour of HH samples is presented. All specimens demonstrate a characteristic increase in force up to the maximum value, after which the load drops rapidly, indicating an abrupt and brittle type of failure. Numerical values of the maximum force are summarised in Table 1, while the corresponding deflections at peak load are listed in Table 2. The curves show limited plastic deformation prior to fracture, suggesting that the hexagonal structure reduces the capacity to redistribute stresses once local failure initiates.

Compared with solid samples, HH specimens exhibit noticeably lower peak forces and fracture at similar or slightly lower deflections, which implies a reduced load–bearing capability. The visible sharp force decline after reaching F_max indicates limited energy absorption and confirms the brittle nature of the failure process. This behaviour may result from stress concentration at the cell walls and joints of the hexagonal lattice, where crack initiation is likely to occur.

Despite reducing the material volume and sample weight, the hexagonal infill negatively affects mechanical strength. The internal architecture likely promotes localized buckling and rapid crack propagation once the critical load is exceeded. Consequently, the HH configuration demonstrates lower structural robustness compared to solid specimens, which should be taken into account in applications where sudden failure is undesirable.

In Figure 3c, the load–deflection curves of the SS specimens are presented. According to Table 1, the highest load capacity was recorded for specimen B_SS_01, while the remaining samples reached slightly lower maximum forces. The corresponding deflection values at peak load are shown in Table 2 and remain within a relatively narrow range, indicating comparable initial stiffness among SS samples. The elevated peak load and higher displacement observed for B_SS_01 suggest improved energy absorption prior to failure, which may result from more uniform stress distribution within the double-spiral architecture. After reaching F_max_, most curves exhibit a force drop associated with structural failure. However, the post-peak behaviour varies noticeably between specimens. Samples such as B_SS_03 and B_SS_04 show an abrupt decrease in load, characteristic of brittle fracture, while B_SS_05 and B_SS_08 demonstrate a more gradual decline, which implies delayed failure progression and the capability to dissipate energy after initial crack initiation. This indicates that although the SS configuration generally ensures higher bending resistance compared to HH structures, its mechanical response remains sensitive to local imperfections and geometry-related stress concentration zones. The Spiral–Spiral design enhances flexural capacity relative to other cellular layouts but introduces variability in deformation mechanisms. Samples capable of maintaining load beyond the peak may be advantageous in applications requiring structural resilience and controlled failure rather than sudden collapse. Samples B_SS_01, B_SS_05, B_SS_06 and B_SS_08 show the most gradual decrease in force after reaching the maximum value, indicating their higher mechanical properties compared to other samples.

Figure 3d shows the results of the bending test for samples with a quasi-self-similar-quasi-self-similar (QQ) structure. The curves in the graph show the phase of linear force increase, the maximum force value, and the force decrease. In the initial phase, up to a deflection of approximately 5 mm, the curves are quite similar, indicating similar initial stiffness of all QQ samples. The maximum force is reached at a deflection of 5 to 10 mm. Samples B_QQ_07 and B_QQ_09 show the highest maximum force values, suggesting significantly higher mechanical properties of these configurations. The force decrease phase after reaching the maximum value varies significantly between samples. Some samples, such as B_QQ_05 and B_QQ_08, show a gradual decrease in force, while others, such as B_QQ_02 and B_QQ_06, have sharp decreases. Sudden decreases in force may be caused by cracks or damage to the internal structure, which is typical for cellular materials. In summary, QQ structures show significant variation in mechanical properties, which may be due to differences in internal cellular structure and sample preparation. These differences affect the maximum strength and the way the material behaves after reaching maximum force.

**Figure 4:**
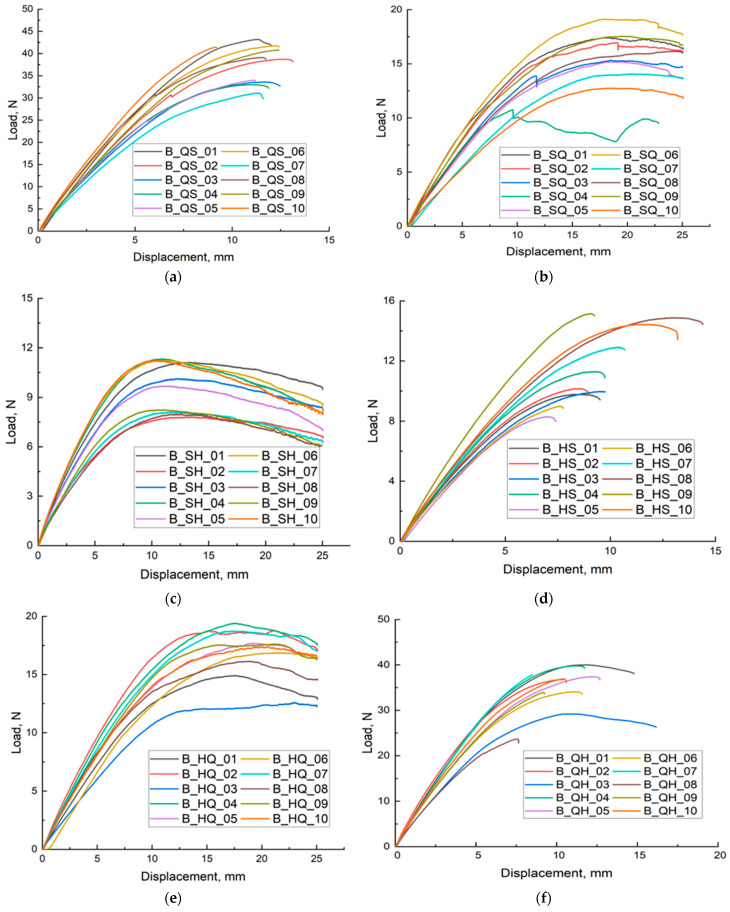
Load–Displacement plots for three-point bending for models manufactured from RGD720 material with the following structures: (**a**) QS; (**b**) SQ; (**c**) SH; (**d**) HS; (**e**) HQ; (**f**) QH.

Figure 4a shows the results of the bending test for samples with a spiral–quasi-self-similar (SQ) structure. Ten curves are labelled B_SQ_01 to B_SQ_10. The force–deflection curves for the SQ structure show an initial increase in force with deflection, reaching a certain maximum, after which the force begins to decrease or remains constant. This may suggest microstructural damage or changes in stress distribution. The maximum force is approximately 18 N, and the deflection reaches 25 mm, suggesting that SQ structures are more flexible than QS structures but less resistant to loads. There are visible differences between the individual curves, which may be due to heterogeneity in the cellular structure or differences in the sample production process.

Figure 4b shows the force–deflection curves for samples with a quasi-self-similar–spiral (QS) cellular structure. The force–deflection curves for QS samples show an increasing trend, suggesting that as the deflection increases, the force also increases. After reaching a certain point, the curves begin to stabilise or show slight deviations, which may be the result of microstructural changes in the material or the onset of local damage. The curves indicate a force of up to approximately 45 N and a deflection of up to 15 mm, suggesting that QS structures are capable of withstanding significant loads before reaching their strength limit. There are some differences between the curves, which may be due to natural variations in the cellular structure of individual samples or material heterogeneity.

Figure 4c shows the bending test results for samples with a spiral–hexagonal (SH) cellular structure. As with QS, each of the ten curves corresponds to one sample, labelled B_SH_01 to B_SH_10. The curves for the SH structure show an initial increase in force with deflection, reaching a maximum, after which the force begins to decrease. This decrease may be the result of structural damage or the onset of sample failure. The force reaches a maximum of approximately 12 N, and the deflection reaches 25 mm, indicating that SH structures can withstand lower loads than QS but can deflect more before complete failure. The curves for the individual samples differ slightly, which may be due to differences in internal structure or material defects.

Figure 4d shows the results of three-point bending for samples with a hexagonal–spiral structure, labelled B_HS_01 to B_HS_10. These samples have a hexagonal structure inscribed on a 10 mm wall and a second spiral structure made on a 6 mm wall, which is marked as HS. These samples were also filled with moulding silicone. Sample B_HS_01 shows an increase in force to approximately 11 N at a deflection of approximately 13 mm, followed by a gradual decrease in force. Sample B_HS_02 reaches a maximum force of approximately 12 N at a deflection of approximately 14 mm, followed by a gradual decrease in force. B_HS_03 reaches a maximum force of approximately 12 N at a deflection of approximately 14 mm, followed by a gradual decrease in force. Sample B_HS_04 reaches a maximum force of approximately 13 N at a deflection of approximately 14 mm, followed by a gradual decrease in force. B_HS_05 reaches a maximum force of approximately 12 N at a deflection of 14 mm, followed by a gradual decrease in force. Samples with a hexagonal–spiral structure exhibit maximum forces in the range of 11–13 N at a deflection of 13–14 mm. Compared to samples with a hexagonal structure, these samples show a more gradual decrease in force after reaching maximum values, suggesting more flexible behaviour of the material. The hexagonal–spiral (HS) structure may contribute to the increased deformation capacity of the sample, which is evident in the test results. The filling with moulding silicone probably contributes to greater energy absorption, which may result in a more gradual decrease in force after reaching maximum values. All samples with a hexagonal–spiral (HS) structure exhibit similar behaviour, suggesting uniformity in their structure and mechanical behaviour. Despite lower maximum forces compared to solid samples, these samples show the ability to undergo greater deformation before fracture, which may be advantageous in applications requiring greater material flexibility.

Figure 4e shows the results of a static bending test for samples with a hexagonal–quasi-self-similar (HQ) structure. The graph shows the relationship between force and deflection for ten samples with different configurations. The general shape of the curves indicates the characteristic phases of material behaviour during bending: the initial linear stage, maximum force and force decline phase. In the initial phase, where the deflection ranges from 0 to approximately 5 mm, all samples show an almost linear increase in force, indicating elastic behaviour of the material. In this range, the curves for all samples are quite similar, suggesting that HQ structures have similar initial stiffness. As the deflection increases further, the curves begin to diverge, reaching different maximum force values. The highest maximum force was observed for sample B_HQ_07, suggesting that this configuration had the best mechanical properties compared to the other samples. After reaching the maximum force, the curves exhibit different behaviours during the force decay phase. Some samples, such as B_HQ_07 and B_HQ_08, have rapid force decay, which may indicate sudden fractures or damage to the internal structure. In summary, HQ structures exhibit diverse mechanical properties, and differences in maximum force values may be due to slight differences in internal structure and sample fabrication.

Figure 4f shows the results of the bending test for samples with a quasi-self-similar hexagonal (QH) structure. As in the case of HQ structures, the graphs showed three characteristic phases of the bending test, which include a linear increase in force, reaching a maximum value, and a sharp decrease in force as a result of the sample breaking. In the initial phase of force increase, the deflection is up to approximately 5 mm, and the curves for all samples are similar, suggesting similar initial stiffness of QH structures. The maximum force is reached in the deflection range of 5 to 10 mm. Samples B_QH_07 and B_QH_09 show the highest maximum force values, suggesting that these configurations have the best mechanical properties. After reaching the maximum force, some samples show a gradual decrease in force, while others, such as B_QH_02 and B_QH_04, have more rapid decreases. The sharp drops may be caused by sudden damage to the internal structure, which is typical for cellular materials subjected to high loads. In summary, QH structures also exhibit a variety of mechanical properties, with the greatest differences observed in the maximum force value and the manner in which the force decreases after reaching its maximum value. Table 1, which presented the maximum forces for solid beams (FF) and nine combinations of cellular structures filled with moulding silicone for standardised structures, shows that the highest force value was 151.32 N and the lowest was 117.96 N. The average values of the maximum forces are significantly lower, which shows that the QS, QQ, QH, HS, SS and HH structures, despite being filled with silicone material, were not able to be carried out in most cases for a deflection of 25 mm. Taking into account the average values among the silicone-filled cellular structures, the highest average value was found for QS structures and amounted to 37.66 N, while the lowest average values were determined for HH and SH structures, which are 9.81 N and 9.69 N, respectively. Taking into account the other values determined, the cellular structures show significant variability in the results presented, which indicates differences in stress distribution that may result from the geometry of the structure, the print of the cellular structure and the material configuration. Direct comparison of the load–displacement curves and derived flexural properties revealed that the fully dense Solid configuration exhibited the highest bending stiffness and peak load, whereas the silicone-filled QS-Silicone variant achieved the best combination of compliance and energy absorption among all cellular structures.

Table 2 presented an analysis of beam displacement under maximum load, showing clear differences in the behaviour of individual geometric configurations. The recorded load–displacement curves enabled quantitative evaluation of flexural stiffness, maximum deflection at peak load, and post-peak ductility, revealing the transition from brittle failure in the fully dense Solid beams to highly compliant, pseudo-ductile behaviour in all silicone-infiltrated cellular configurations. The average deflection values ranged from 9.37 mm to 19.78 mm. The largest displacement was obtained for the HQ beam (19.78 mm), which indicates its highest susceptibility to deformation under bending conditions. The SQ beam (18.47 mm) showed similar susceptibility, also characterised by the highest standard deviation of 3.66 mm, which suggests significant variation in the behaviour of the samples in this configuration. Relatively high deflection values were also recorded for the FF beam (15.78 mm) and QQ beam (14.24 mm). The lowest displacement values occurred for the HS beams (9.37 mm) and QH beams (10.31 mm), which can be considered the most rigid among the analysed samples. Standard deviation values ranging from 0.68 to 2.07 mm confirm the moderate repeatability of the results in most cases, with the greatest stability obtained for the FF beam. A comparison with the sample volumes indicates that greater deflections characterised beams with larger volumes, such as FF (7.20 cm^3^), HQ (2.57 cm^3^) and SQ (3.55 cm^3^), while samples with smaller volumes, such as HH (1.98 cm^3^) and HS (2.81 cm^3^), showed lower displacements, which is consistent with the relationship between geometric stiffness and material volume in cross-section.

The bending work (W) was calculated using the classical formula, by directly multiplying force (F) by displacement (s):

W = Fs(1)

This approach reflects the basic mechanical interpretation of work as the action of a force over a given displacement. The fully dense Solid reference beams exhibited the highest mean deformation work of 2.15 ± 0.16 J, owing to the high stiffness and strength of the rigid RGD720 photopolymer. All silicone-filled cellular specimens displayed substantially lower values, ranging from 0.11 ± 0.02 J (HH-Silicone and HS-Silicone) to 0.43 ± 0.06 J (QS-Silicone), corresponding to a 5- to 20-fold reduction compared to the solid beam. This marked decrease is primarily a consequence of replacing the stiff photopolymer with the compliant silicone, which dominates the mechanical response of the infiltrated lattices. Among the silicone-filled configurations, the highest energy absorption was achieved by structures combining larger effective silicone volume with more deformable unit cells, namely QS-Silicone (0.43 ± 0.06 J), QQ-Silicone (0.42 ± 0.05 J), and QH-Silicone (0.36 ± 0.09 J). In contrast, honeycomb-based hybrids with the smallest material volume (HH-Silicone, SH-Silicone, HS-Silicone) absorbed only approximately 0.11 J, reflecting their higher relative stiffness within the silicone-filled series.

The coefficients of variation remained low across all groups (typically 4–9% for silicone-filled specimens and 7.4% for FF-Solid), confirming excellent manufacturing repeatability and uniform silicone infiltration. These results highlight the ability to systematically tailor energy absorption at peak load through the selection of unit cell topology and core–skin arrangement, providing a clear trade-off between load-bearing capacity and compliance in silicone-filled hybrid lattices. Full numerical data for all ten replicates of each configuration are presented in Table 3.

**Figure 5:**
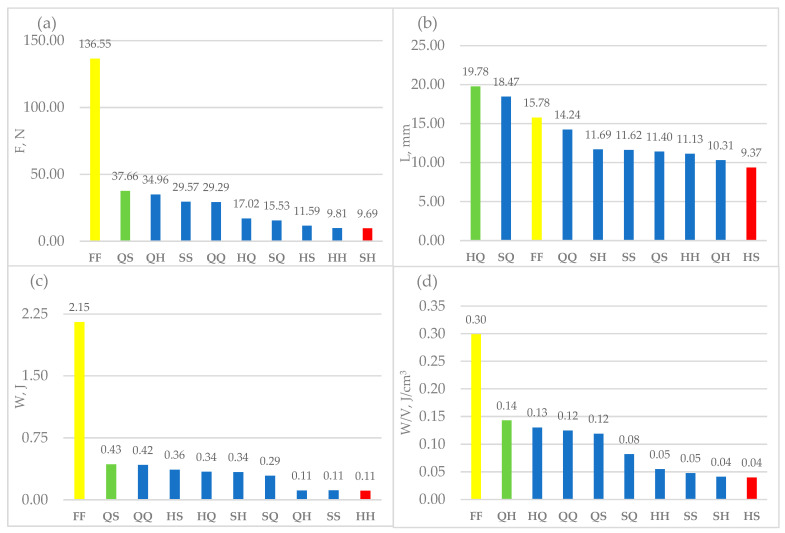
Mechanical response comparison of cellular architectures relative to solid reference: (**a**) Maximum Force, (**b**) Displacement at Maximum Force, (**c**) Work to Failure, (**d**) Energy per Volume.

A comparative evaluation of averaged mechanical responses across all cellular configurations (Figure 5a–d) demonstrates that topological modification of the solid baseline induces measurable and directionally consistent trends in strength, deformation response and energy management. The influence is geometry-dependent rather than purely porosity-driven.

In Figure 5a (Maximum Force), all architected structures exhibit a reduction in peak load relative to the dense reference; however, the scale of degradation varies markedly between topologies. This confirms that load-transfer efficiency is controlled primarily by internal cell architecture, where continuous strut connectivity mitigates stress concentration, while discontinuous layouts accelerate failure. In Figure 5b (Displacement at Maximum Force), several cellular configurations reach significantly higher deflections than the reference. Increased displacement indicates reduced initial stiffness and a more ductile-like bending mode, enabling larger deformation before fracture. Variants marked in green represent the most compliant architectures, whereas red-marked designs correspond to premature failure and brittle response. For Figure 5c (Work/Energy to Failure), a global decreasing tendency is observed, yet selected structures retain notably favourable energy absorption capacity despite reduced material volume. This suggests that appropriately oriented cell paths enhance crack deflection and extend load redistribution time, improving overall failure resistance. When normalized to volume in Figure 5d (Energy per Volume), differences between topologies become more pronounced. High-performing configurations (green) achieve superior mass-efficiency, providing high energy dissipation relative to material usage. Conversely, low-performing variants (red) deliver limited energy capacity per volume, reducing suitability for lightweight structural applications.

Overall, Figure 5a–d collectively confirm that cellular architectures are not merely weakened solids, but design frameworks enabling targeted tuning of stiffness, strength and energy behaviour. Depending on internal geometry, structures may be optimised for rigidity, flexibility or energy management, allowing mechanical performance to be tailored to application-specific functional criteria.

## 4. Discussion

The maximum bending force results obtained (Table 1) indicate a clear variation in the load-bearing capacity of the samples depending on the type of cellular structure and its distribution. The highest force values were recorded for the solid sample (FF), reaching an average of 136.55 N, which is consistent with the predictions for a solid material with a homogeneous cross-section. The use of cellular structures leads to a reduction in flexural stiffness, but in many cases a favourable compromise was achieved between weight reduction and load-bearing capacity, which remained within the range of 30–40% compared to the solid sample. In the group of structures with a hexagonal component (H), average maximum forces of 9–11 N were recorded for homogeneous systems (HH) and 12–19 N for mixed systems (HQ, HS, QH). These values are similar to the data published by Yazıcı et al. [27], where honeycomb structures exhibited 20–25% of the load-bearing capacity of a solid sample in three-point bending. This means that photopolymerisable structures with hexagonal geometry retain a comparable level of flexural strength to their polymer counterparts manufactured by using the FDM method.

Structures with a quasi-self-similar (Q) component, such as QQ, QS, SQ and QH, were characterised by significantly higher maximum force values—in the range of 30–40 N, with little variation in standard deviation (4–5 N). This behaviour can be attributed to the multi-scale arrangement of load-bearing elements, which results in a more favourable stress distribution during bending. A similar effect was described in [28], where re-entrant and quasi-fractal structures showed an increase in effective stiffness in the direction perpendicular to the printing plane. Spiral structures (S), both in homogeneous (SS) and mixed (SH, SQ, QS) configurations, showed average bending forces of 26–30 N, which corresponds to approximately 20–22% of the strength of the full sample. Unlike hexagonal systems, the deformation of these structures is more continuous and free of local buckling, suggesting the involvement of a rib bending mechanism rather than local compression. When analysing data variability, it should be noted that mixed structures (e.g., QS, QH, SQ) achieved the most favourable load-bearing capacity to dispersion ratio, indicating stable load transfer. This suggests that systems combining different topologies can be an effective solution in the design of elements with varying local stiffness. Further research should include an analysis of the impact of wall thickness and printing direction in the context of stiffness gradient in the cross-section area. It is also advisable to combine experimental measurements with numerical analysis (e.g., using the finite element method) in order to determine the stress distribution in composite structures.

The FF beam, with the largest volume of 7.20 cm^3^ and no silicone additive, had the highest load capacity in the three-point bending test, achieving an average maximum force of 136.55 N. At the same time, it showed the highest deformation work (2.15 J) and displacement at maximum load of 15.78 mm, indicating its ability to both transfer loads and store elastic energy. All other beams containing silicone exhibited significantly lower load-bearing capacity. For example, the HH and SH beams achieved an average of 9.81 N and 9.69 N, respectively, which is less than 10% of the value obtained for FF, and their displacement under maximum load ranged from 9.37 mm (HS) to 18.47 mm (SQ), often exceeding the value of FF due to the greater flexibility introduced by silicone. The energy storage capacity of these samples was also limited, with an average deformation work of 0.11–0.43 J, which is significantly lower than that of the FF beam.

A clear correlation was observed between the actual printed volume of the silicone-filled lattices and their energy absorption up to peak load. Configurations with the largest silicone volume (SS-Silicone: 7.11 cm^3^ and QS-Silicone: ≈3.6 cm^3^) exhibited the highest absorbed energy within the infiltrated series, reflecting the greater amount of compliant elastomer available to deform. In contrast, the smallest-volume honeycomb-based structures (HH-Silicone: 1.98 cm^3^ and similar hybrids) displayed the lowest energy values, confirming that below a critical material volume the soft filling contributes negligibly to overall deformation work.

Hybrid core–skin architectures (QS-, SQ-, QH-Silicone, etc.) consistently showed intermediate stiffness and notably low coefficients of variation in both peak force and absorbed energy. This improved repeatability is attributed to more uniform stress distribution and reduced sensitivity to local defects at the resin–silicone interface compared with uniform lattices. Consequently, deliberate combination of different unit cell topologies in the core and skin layers offers an effective design strategy for achieving a predictable balance between load-bearing capacity, flexibility, and energy dissipation in polymer–elastomer hybrid beams.

Analysis of the results confirms that the presence of silicone in the beams increased flexibility and displacement under load, but at the cost of a significant reduction in maximum load-bearing capacity and deformation energy compared to the FF sample. Overall, the FF beam served as a reference point in terms of strength and energy accumulation capacity, while the silicone samples showed a compromise between flexibility and strength, depending on the volume and geometry of the individual configurations.

Among the silicone-filled cellular beams investigated, the QS-Silicone configuration consistently demonstrated the best compromise between peak load (highest among lattices), total deformation capacity, and absorbed energy up to peak force. This superior performance is attributed to the combination of a compliant quasi-similar core that permits large elastic buckling and energy dissipation in the silicone phase, with spiral skin layers that provide enhanced in-plane stiffness and more uniform stress transfer from the loading roller to the lattice interior.

The results confirm that infiltration of open-cell photopolymer lattices with a soft elastomer dramatically increases ductility and failure tolerance, yet at the cost of a 5- to 20-fold reduction in load-bearing capacity and energy absorbed to peak load compared with the fully dense Solid reference. Consequently, silicone-filled cellular beams are unsuitable as direct structural replacements for solid components under high bending loads, but offer attractive properties where controlled compliance, vibration damping, or progressive energy dissipation are prioritised over absolute strength.

These findings highlight the importance of unit-cell topology and core–skin arrangement in tailoring the mechanical response of polymer–elastomer hybrids produced by PolyJet technology. Future work could explore graded silicone filling, selective reinforcement of critical zones with rigid photopolymer, or multi-material gradient designs to approach more closely the strength–toughness envelope of the fully dense material while retaining the benefits of cellular architecture.

## 5. Conclusions

In the analysis of silicone-filled cellular structures under quasi-static three-point bending, the quasi-self-similar–spiral (QS) configuration emerges as the most mechanically advantageous solution. QS specimens demonstrate one of the highest mean maximum loads among all cellular configurations (F = 37.66 N) while maintaining moderate standard deviation (SD = 4.34 N), indicating both high load-bearing capacity and reliable structural response. The corresponding energy absorption (W = 0.43 J) and displacement at maximum load (L = 11.40 mm) confirm that QS efficiently distributes stresses along the beam length, achieving a balance between stiffness, deformability, and repeatability. Importantly, QS maintains these mechanical properties at a relatively low material volume (V = 3.61 cm^3^), approximately half that of the fully solid reference sample (FF: V = 7.20 cm^3^, F = 136.55 N, W = 2.15 J), demonstrating a favourable trade-off between strength, durability and material efficiency. The fully solid FF sample serves as a benchmark, providing maximum load and energy absorption; however, its large volume and weight limit suitability for lightweight applications. Among cellular variants, the SS structure exhibits high mechanical stability, maintaining consistently high minimum forces with low variability, which indicates a predictable structural response. Although SS does not reach the peak force of QS, its reliability may be beneficial where uniform deformation is required.

Hexagonal-based configurations (HH, HS, SH) show lower strength and energy absorption, but HH demonstrates excellent repeatability despite limited maximum forces. Quasi-self-similar motifs implemented in QH and QQ structures increase stiffness relative to purely geometric patterns, although they introduce greater variability in the force–deflection response, emphasising the need for precise silicone distribution.

Overall, the mechanical performance of silicone-filled beams strongly depends on both structural motif and effective material volume. The QS configuration provides the most balanced compromise between load capacity, displacement, energy absorption and material efficiency, making it the most versatile option among the tested cellular geometries. Integration of various cellular motifs with controlled silicone filling offers a promising strategy for tailoring mechanical behaviour and optimising performance for specific engineering applications.

## Figures and Tables

**Table 1:** Maximum load determined for three-point bending.

No	Maximum Load (F), N									
	FF	HH	QQ	SS	QS	SQ	SH	HS	HQ	QH
1	137.49	10.42	33.29	33.80	43.19	17.43	11.11	9.80	14.93	40.02
2	151.32	8.85	36.23	25.96	38.72	16.96	7.82	10.17	18.78	36.78
3	144.78	9.31	22.81	25.73	33.59	15.33	10.12	9.98	12.60	29.20
4	130.10	11.47	27.40	28.23	32.99	10.76	11.33	11.30	19.40	39.82
5	143.38	9.27	22.81	29.39	33.99	15.18	9.69	8.29	17.67	37.40
6	131.10	9.62	28.85	31.79	41.73	19.13	1.,24	8.99	16.87	34.08
7	117.96	9.07	35.88	30.63	31.05	14.06	8.09	12.90	18.73	37.80
8	125.66	9.41	26.81	31.88	39.10	16.16	7.98	14.88	16.14	23.68
9	140.43	10.82	29.33	27.79	40.77	17.55	8.24	15.14	17.63	33.93
10	143.26	9.86	29.45	30.50	41.44	12.77	11.23	14.43	17.41	36.92
$\bar{X}$	136.55	9.81	29.29	29.57	37.66	15.53	9.69	11.59	17.02	34.96
SD	10.16	0.84	4.73	2.64	4.34	2.49	1.52	2.56	2.04	5.08
Max	151.32	11.47	36.23	33.80	43.19	19.13	11.33	15.14	19.40	40.02
Min	117.96	8.85	22.81	25.73	31.05	10.76	7.82	8.29	12.60	23.68

**Table 2:** Determined displacement at maximum force for three-point bending.

No	Displacement (L) at Maximum Load (F), mm									
	FF	HH	QQ	SS	QS	SQ	SH	HS	HQ	QH
1	16.46	10.18	13.75	10.31	11.26	18.40	13.27	8.23	17.54	11.77
2	16.46	16.46	16.46	12.88	12.59	18.99	13.49	8.34	21.12	10.25
3	15.26	10.35	14.20	13.57	11.63	18.54	12.24	9.52	22.90	10.98
4	16.60	11.13	15.34	11.29	10.93	9.59	10.86	9.32	17.54	11.13
5	15.10	10.93	14.20	12.41	11.11	18.43	11.06	7.00	19.32	12.25
6	15.65	10.28	12.85	10.79	12.18	17.88	10.90	7.58	21.35	11.05
7	16.66	11.56	13.33	12.25	11.26	20.40	11.52	10.37	17.53	8.50
8	15.27	11.79	16.00	11.18	11.54	24.53	12.55	12.98	18.90	7.56
9	15.13	8.73	12.50	11.41	12.35	19.40	10.57	9.02	21.08	9.17
10	15.19	9.92	13.78	10.16	9.16	18.48	10.40	11.34	20.50	10.42
$\bar{X}$	15.78	11.13	14.24	11.62	11.40	18.47	11.69	9.37	19.78	10.31
SD	0.68	2.07	1.31	1.12	0.97	3.66	1.13	1.80	1.90	1.48
Max	16.66	16.46	16.46	13.57	12.59	24.53	13.49	12.98	22.90	12.25
Min	15.10	8.73	12.50	10.16	9.16	9.59	10.40	7.00	17.53	7.56

**Table 3:** Absorbed energy up to peak load in three-point bending.

	FF	HH	QQ	SS	QS	SQ	SH	HQ	HS	QH
V, cm^3^	7.20	1.98	3.37	7.11	3.61	3.55	2.74	2.57	2.81	2.55
No	**Work/Energy (W/E), J**									
1	2.26	0.11	0.46	0.34	0.49	0.32	0.15	0.26	0.08	0.47
2	2.49	0.15	0.43	0.47	0.49	0.32	0.11	0.40	0.08	0.38
3	2.21	0.10	0.37	0.31	0.39	0.28	0.12	0.29	0.10	0.32
4	2.16	0.13	0.43	0.31	0.36	0.10	0.12	0.34	0.11	0.44
5	2.16	0.10	0.42	0.28	0.38	0.28	0.11	0.34	0.06	0.46
6	2.05	0.10	0.41	0.31	0.51	0.34	0.12	0.36	0.07	0.38
7	1.97	0.10	0.41	0.44	0.35	0.29	0.09	0.33	0.13	0.32
8	1.92	0.11	0.51	0.30	0.45	0.40	0.10	0.31	0.19	0.18
9	2.12	0.09	0.35	0.33	0.50	0.34	0.09	0.37	0.14	0.31
10	2.18	0.10	0.42	0.30	0.38	0.24	0.12	0.36	0.16	0.38
$\bar{X}$	2.15	0.11	0.42	0.34	0.43	0.29	0.11	0.34	0.11	0.36
SD	0.16	0.02	0.05	0.06	0.06	0.08	0.02	0.04	0.04	0.09
Max	2.49	0.15	0.51	0.47	0.51	0.40	0.15	0.40	0.19	0.47
Min	1.92	0.09	0.35	0.28	0.35	0.10	0.09	0.26	0.06	0.18

## Data Availability

The original contributions presented in this study are included in the article. Further inquiries can be directed to the corresponding author.
